# Clinical assessment of effusion in knee osteoarthritis—A systematic review

**DOI:** 10.1016/j.semarthrit.2015.10.004

**Published:** 2016-04

**Authors:** Nasimah Maricar, Michael J. Callaghan, Matthew J. Parkes, David T. Felson, Terence W. O׳Neill

**Affiliations:** a Arthritis Research UK Centre for Epidemiology, Institute of Inflammation and Repair, Faculty of Medical and Human Sciences, Manchester Academic Health Science Centre, University of Manchester, Manchester M13 9PT, UK; b NIHR Manchester Musculoskeletal Biomedical Research Unit, Central Manchester NHS Foundation Trust, Manchester Academic Health Sciences Centre, Manchester, UK; c Clinical Epidemiology Unit, Boston University School of Medicine, Boston, MA

**Keywords:** Clinical, Tests, Knee, Effusion, Osteoarthritis, Reliability, Sensitivity, Specificity

## Abstract

**Objective:**

The aim of this systematic review was to determine the validity and inter- and intra-observer reliability of the assessment of knee joint effusion in osteoarthritis (OA) of the knee.

**Methods:**

MEDLINE, Web of Knowledge, CINAHL, EMBASE, and AMED were searched from their inception to February 2015. Articles were included according to a priori defined criteria: samples containing participants with knee OA; prospective evaluation of clinical tests and assessments of knee effusion that included reliability, sensitivity, and specificity of these tests.

**Results:**

A total of 10 publications were reviewed. Eight of these considered reliability and four on validity of clinical assessments against ultrasound effusion. It was not possible to undertake a meta-analysis of reliability or validity because of differences in study designs and the clinical tests. Intra-observer kappa agreement for visible swelling ranged from 0.37 (suprapatellar) to 1.0 (prepatellar); for bulge sign 0.47 and balloon sign 0.37. Inter-observer kappa agreement for visible swelling ranged from −0.02 (prepatellar) to 0.65 (infrapatellar), the balloon sign −0.11 to 0.82, patellar tap −0.02 to 0.75 and bulge sign kappa −0.04 to 0.14 or reliability coefficient 0.97. Reliability and diagnostic accuracy tended to be better in experienced observers. Very few data looked at performance of individual clinical tests with sensitivity ranging 18.2–85.7% and specificity 35.3–93.3%, both higher with larger effusions.

**Conclusion:**

The majority of unstandardized clinical tests to assess joint effusion in knee OA had relatively low intra- and inter-observer reliability. There is some evidence experience improved reliability and diagnostic accuracy of tests. Currently there is insufficient evidence to recommend any particular test in clinical practice.

## Introduction

Knee effusion is common among people with knee osteoarthritis (OA). In those with knee pain and having radiographic OA, nine out of 10 people will have imaging evidence of effusion with 55% having a moderate to large effusion [1]. Clinical assessment for knee effusion is thus an integral part of routine physical examination in knee OA. A range of clinical tests have been used to assess the presence of knee effusion including visible inspection of swelling [2], palpation of the knee [3], [4], and a range of dynamic tests including ballottement, patellar tap and the sweep test, though the terminology used in the literature to describe the tests is inconsistent [2], [4], [5], [6], [7]. To have clinical value and utility, a clinical test should be both valid, in that it should detect an effusion if present and exclude it if not present, and also reliable, meaning that repeated assessments with either the same or different observers result in the same conclusion. Knowledge of the reliability and validity of the currently used clinical tests for knee effusion in knee OA would help inform the optimum approach taken in clinical assessment of these patients and help in informing diagnostic or intervention strategies. To the best of our knowledge there has been no systematic review that pool reliability and validity data from individual studies. We therefore undertook a systematic review with the aim of determining (1) intra- and inter-observer reliability and (2) performance characteristics, of clinical tests for the presence of effusion in knee OA.

## Methods

### Search strategies

Publications which reported either intra- and inter-observer reliability of clinical assessments of knee effusion or the validity of clinical assessments using imaging such as ultrasound (US) and magnetic resonance imaging (MRI) were identified from searching six databases up to February 2015: Medline (1948 onwards), Embase (1974 onwards), AMED (1985 onwards), Cumulative Index to Nursing and Allied Health Literature (CINAHL) Plus (1937 onwards), Web of Knowledge (1950 onwards), and the Cochrane Central Registers for Controlled Trials. The databases were searched individually for all possible terms and combination of terms to accommodate differences in their search engines. All medical subject-heading searches (MeSH) were exploded when possible. The key terms used in combination (“OR”) were test, examination, assessment, physical, clinical, MRI, US, effusion and swelling which were then used in combination (“AND”) with knee. Full details of the MEDLINE search strategy appear in Appendix 1. Hand searches were also performed in addition to additional searches through Google Scholar and Reference Manager Search engines. Reference lists of publications were also searched for other relevant publications. There was no language restriction. Eligibility assessment of trials for inclusion in the review was performed unblinded by 1 reviewer (NM).

### Study selection criteria

Publications considered were those that included adults with knee OA based on the American College of Rheumatology (ACR) clinical classification criteria for OA [8] or from detailed clinical and/or radiographic assessment of the knee joint. Due to a limited number of studies on knee OA, for this review, studies with mixed samples of OA and inflammatory arthritis (IA) or knee pain were also included. Studies were selected if they reported evaluation of the validity of clinical assessment of knee effusion against imaging assessment of effusion, or the reliability of clinical assessment of effusion undertaken either by the same observer (intra-observer reliability) or different observers (inter-observer reliability).

### Quality assessment

For publications evaluating reliability of the clinical tests for knee joint effusion, study quality was graded using the “reliability” section of COnsensus-based Standards for the selection of health Measurement INstruments (COSMIN) with close reference to a separate publication which provided detailed description of the scoring system [9]. To evaluate the quality of diagnostic accuracy studies on knee joint effusion, the diagnostic study appraisal checklist from Centre of Evidence-Based Medicine (CEBM) was used. Two assessors (NM, MJC) independently assessed and scored the publications for quality and reached consensus in cases of disagreement.

### Data extraction and analyses

The data from the selected papers were extracted independently by two reviewers (NM, MJP) using a standardised form. Information about the type of study, methods evaluated, subject and observer characteristics, assessment protocols and their results including relevant data for calculation of sensitivity, specificity and reliability of the effusion tests were extracted. In this review, “sensitivity” refers to the ability of a test to identify correctly the presence of effusion in individuals with knee effusion and “specificity” the ability of the test to correctly exclude individuals without knee effusion, assessed by an US or MRI as “gold standard.” Intra- and inter-observer reliability is a measure of the degree of agreement when the test is repeated by the same observer or between different observers, respectively. The kappa (κ) statistic was used to denote agreement for dichotomous variables while weighted kappa (κ^ω^) and reliability coefficient (R_c_) was used for ordinal and continuous variables, respectively. We did not undertake a formal meta-analysis for reliability and performance characteristics data because of the different methodologies and differences in reporting among the studies. For kappa and R_c_, we considered values of less than 0.2 to indicate poor agreement, between 0.21 and 0.40 fair, 0.41–0.60 moderate, 0.61–0.80 as good and values more than 0.80 to indicate excellent agreement [10].

## Results

### Search outcome

**Fig. 1:**
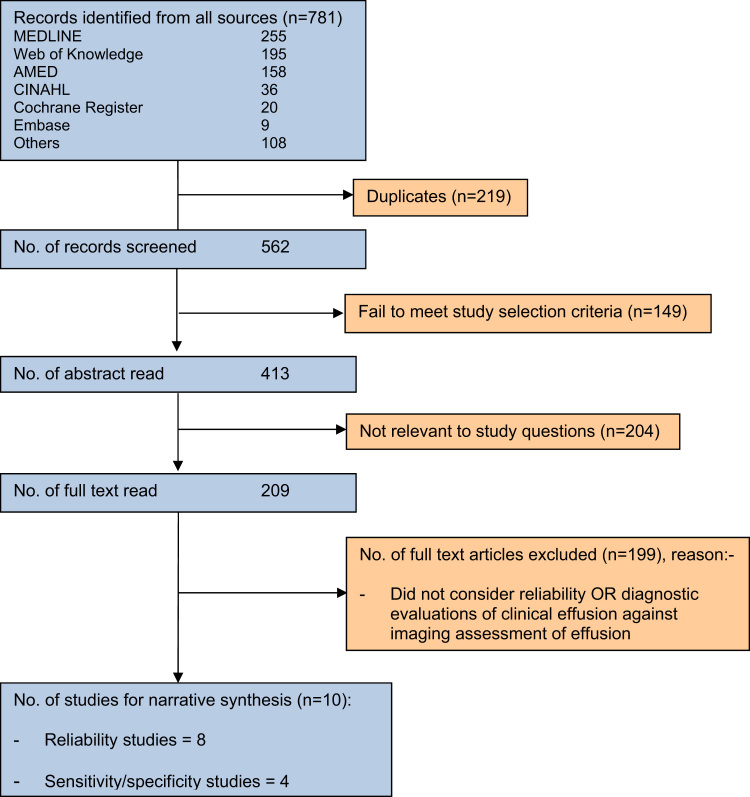
Article search and selection process.

The results of the search are summarized in Figure 1. In total 10 publications met the inclusion criteria; some of these addressed intra- and/or inter-observer reliability only, others reliability and validity (Table 1, Table 2). Several authors were contacted for further information. Where information was obtained it was included in the review [11], [12]. Two articles reported results from the same trials and were considered as one study to avoid potential bias from over-reporting/counting [11], [13].

### Characteristics of included studies

Eight publications evaluated intra- and/or inter-observer reliability testing for clinical tests of effusion [12], [14], [15], [16], [17], [18], [19], [20]. The number of assessors varied from two to six [12], [14], [15], [16], [17], [18], [19], [20]. Two studies reported on reliability of skilled rheumatologists, orthopedic specialists and medical consultants [14], [18], four on clinicians of mixed experience such as trainees and consultants [15], [16], [19], [20] and one study reported on reliability of physiotherapists with 7–10 years of post-qualification experience [12].

Four publications reported sensitivity and/or specificity of knee effusion tests where the clinical tests were compared against US as the standard [11], [18], [20], [21]. We found one study [22] that compared clinical assessment of effusion against MRI assessment. However, we were unable to include this study as it did not provide sufficient data to allow the determination of sensitivity and specificity and also the clinical test used to assess the knee effusion was not stated. Of the 10 publications included in the review, seven comprised patients with knee OA [11], [14], [15], [16], [19], [20], [21], one comprised patients with knee pain (which included those with OA and inflammatory arthritis) [18], one recent presentation to primary care or to rheumatology clinics or on the waiting list for total knee replacement that included patients with a range of knee pain severity and OA [12], and one comprised a population sample including people with knee OA [17].

### Clinical tests used in studies

Clinical tests used to assess knee effusion were categorized as either visual inspection, or dynamic testing involving (1) movement of fluid across the knee with the presence of effusion denoted by reappearance of fluid distension (bulge sign) and (2) pressure over the patella with the presence of effusion determined by palpable ballottement or tapping of the patella against the femoral condyle (patellar tap), or palpable distension of the underlying joint line by fluid fluctuation (balloon sign). We looked also at studies which used combinations of these tests and also when the particular method used was not stated.

Three studies [12], [16], [21] included assessment of visual inspection. A further study included assessment of visual inspection in combination with other clinical tests [18]. Five studies included assessment of the bulge sign [12], [14], [16], [18], [20], with two of these in combination with other tests [18], [20]. Two studies included assessment of “ballottement” [16], [21], two the “patellar tap” [12], [14] and in one ballottement and patellar tap tests were used in combination with bulge sign [20]. Two studies included assessment of “balloon” sign [12], [14] where the test procedure was described in one [12]. Hauzeur et al. [18] included assessment of a combination of tests that included inspection, the bulge sign and a “compression” test as described by Moder and Hunder [23] to differentiate joint effusion from synovial thickening. Joint effusion was diagnosed when after compressing the fluid into the suprapatellar pouch, the edge of the resulting bulge disappeared on release of the compression. One other study appeared to include the observation of a bulge sign within its 4-point grading scale [19]. In two studies the clinical test for effusion was described as “non-bony or soft-tissue swelling” without further elaboration on the clinical test(s) used [15], [17].

### Data quality

**Fig. 2:**
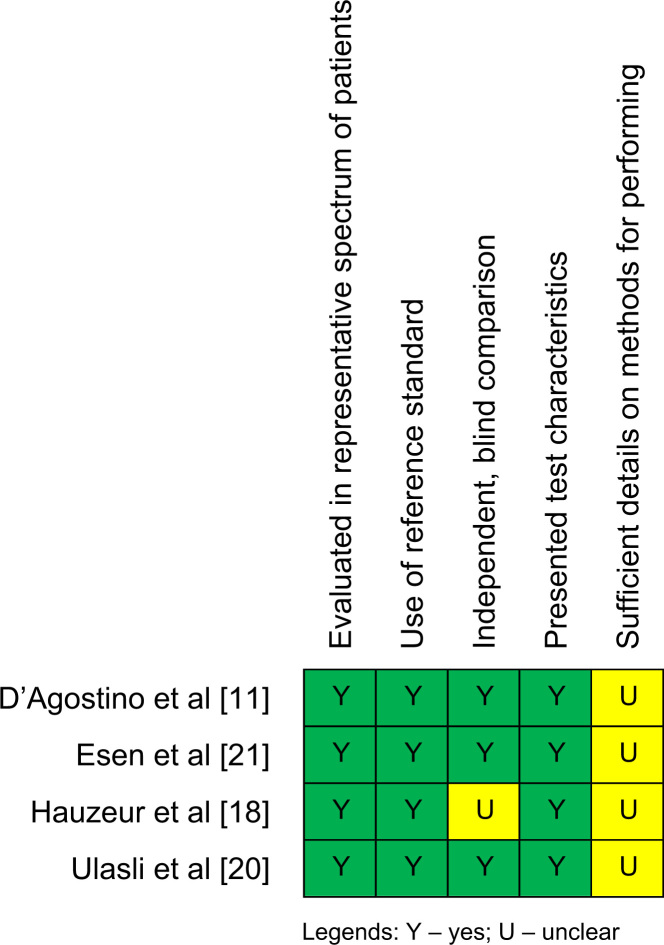
Methodological quality summary on diagnostic studies.

**Fig. 3:**
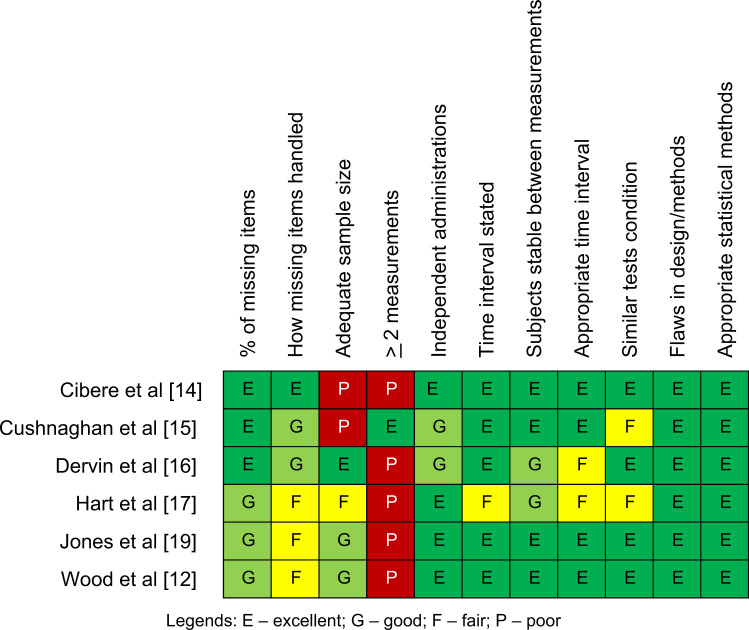
Methodological quality summary on reliability studies.

All studies on validity of clinical tests satisfied the required quality items though three were unclear in one [11], [20], [21] and another was unclear in two quality items [18] (Fig. 2). For the reliability studies, all studies scored as either excellent, good, or fair on quality items with the exception of two items which were rated as poor; if adequate sample size was used [14], [15] and, when a clinical test was performed, whether the test was repeated more than two times during the reliability testing [12], [14], [16], [17], [19] (Fig. 3).

## Reliability of clinical tests of knee joint effusion

### Visual inspection

One study recorded the kappa agreement for visual inspection of effusion at specific areas around the knee [12]. The highest inter-observer agreement was noted for swelling at the infrapatellar site (κ = 0.65), with fair reliability over the medial gutter (κ = 0.28) and poor reliability over the suprapatellar (κ = 0.13) and prepatellar (κ = −0.02) sites. Intra-observer agreement was higher particularly at the prepatellar site (κ = 1), good at the infrapatellar site (κ = 0.66), moderate at the medial gutter (κ = 0.57) and fair at the suprapatellar site (κ = 0.37) [12]. In another study that did not state where the knee visual inspection was carried out, the inter-observer agreement for visible effusion was poor (κ = 0.28); no intra-observer value was provided [16].

### Bulge sign/sweep

Inter-observer agreement was reported as good in one study that used a four-point scale (1 = none, 2 = mild, 3 = moderate, 4 = severe; R_c_ = 0.82) at a pre-standardization stage and excellent (R_c_ = 0.97) when using a dichotomous scale (present/absent) at post-standardization stage [14]. In another study, however, the inter-observer agreement for bulge sign when assessed on a dichotomous scale following post-standardization was found to be poor (κ = −0.04) [12]. Inter-observer agreement was also poor (κ = 0.14) in one other study where the grading was not described [16]. As for intra-observer agreement, when assessed on a dichotomous scale (present/absent) it achieved moderate reliability (κ = 0.47) [12].

### Patellar tap/ballottement

In the three studies that evaluated the ballottement or patellar tap test, one reported good inter-observer agreement (κ = 0.75) [14] while the other two reported poor agreement (κ = 0.19, −0.02) [12], [16]. Intra-observer agreement investigated in one study was found to be poor (κ = −0.03) [12].

### Balloon sign

At post-standardization, the balloon sign scored as absent/present using 6 patients with knee OA was reported to have excellent inter-observer agreement by a group of skilled and experienced rheumatologists (κ = 0.82) [14] but poor (κ = −0.11) by a group of physiotherapists [12]. Wood et al. [12] reported fair intra-observer agreement of the balloon sign performed by a physiotherapist (κ = 0.37).

### Combination tests/unknown

Some studies reported reliability using combinations of approaches [12], [18], [20]. A combination of inspection performed with palpation including the bulge sign and compression test showed moderate inter-observer agreement (κ^ω^ = 0.45) [18] while combination of bulge sign, ballottement and patellar tap tests had fair inter-observer agreement (κ = 0.25) [20]. Intra-observer agreement was not evaluated in these studies. In another study, the bulge sign, balloon test, and inspection over the medial gutter and suprapatellar pouch were used to derive to an overall judgment of effusion and when assessed on a dichotomous scale had fair reliability (κ = 0.39) and on a 4-point scale moderate reliability (κ^ω^ = 0.43) [12]. However, the intra-observer agreement differed little whether a dichotomous (κ = 0.32) or a 4-point scale (κ = 0.35) was used [12]. The inter-observer agreement for non-bony or soft-tissue swelling where the tests used were unclear range from κ = 0.18 to κ = 0.28 while their intra-observer agreement range from κ = 0.67 to κ = 0.91 [15], [17], [19].

## Performance characteristics: Sensitivity and specificity

Due to the ambiguity of the clinical tests used in most studies (Table 2), we could only report on the performance of two individual clinical tests; visual inspection over the suprapatellar area and the ballottement test. In all studies the gold standard was ultrasound effusion appearance. Both tests had poor sensitivity (18.2% and 32.7%) but high specificity (93.3% and 88.9%) for detecting effusion though how these tests were performed and graded was not described [21]. One study performed visual inspection in combination with other clinical tests including the bulge sign and compression and found sensitivity and specificity of detecting effusion increased by effusion size (large effusion 100% and 78% versus moderate effusion 79% and 25%, respectively) [18]. When a combination of bulge sign, ballottement and patellar tap was used, the sensitivity was higher than its specificity [20]. This study also found both sensitivity and specificity of tests increased with seniority of assessor [20]. Another study assessing effusion on a 4-point scale had a sensitivity of 42.5% and specificity of 72.5% [11].

## Discussion

This systematic review found a paucity of data surrounding the diagnostic and reliability of clinical assessment of effusion in knee OA. In this systematic review there was a wide range of reliability in the clinical assessment of knee effusion though overall most unstandardized clinical tests had poor to moderate reliability. When compared to effusion as detected by imaging (US), sensitivity and also specificity of clinical assessment varied substantially. As might be expected a combination of clinical tests improved sensitivity. There was some evidence that clinical experience impacted on reliability of the clinical tests with reliability being higher among more skilled clinicians. There was no good evidence based on either reliability or performance characteristics to favor any individual clinical test.

The variability in reliability is perhaps not surprising and likely due to a range of factors including the use of standardized protocols and standardization of the clinical assessment, clinical experience of those testing for effusion, differences in the baseline prevalence of effusion and perhaps also differences in size of the effusion.

In terms of standardization, two studies included prior training sessions to standardize the clinical examinations [12], [14] though their findings varied. In one [14], changes were made to the examination protocol following formal analyses of reliability and this could have further contributed to the high inter-observer reliability values during the post-standardization stage. The two studies, however, also used different designs and samples that could have led to differences in the findings. Cibere et al. [14] used six rheumatologists who looked at six patients all assessed in a single day, including repeated testing on the same individual by individual assessors raising the possibility of recall bias. Wood et al. [12] assessed pairs of physiotherapists on 58 patients over a 6-month period where the learning effect of standardization may have diminished over time. Additionally, Cibere et al. [14] recruited patients with more severe disease (as determined by the presence of definite radiographic features of OA) than Wood et al. [12] who also included patients who presented to primary care with knee pain (only 14% had OA based on ACR).

Some of the observed variability in reliability assessment may also be due to differences in the baseline frequency of knee effusion. Kappa values are effected by prevalence of the exposure; high or low prevalence in a sample tends to lower the value of kappa despite high percentage total agreement [24]. For example, in the study of Wood et al. [12] the patellar tap had low prevalence of 0.02 with subsequent low kappa value (−0.02) for inter-observer agreement despite a high percentage agreement of 96.5%. Similar findings were also seen for intra-observer agreement for the patellar tap test (prevalence = 0.03) where the kappa value was near null (k = −0.03) while the percentage agreement was high at 93.6% [12]. Also the differences in the size of the effusion may potentially have impacted on the outcomes—we found evidence from at least one study that performance of the test varied by the size of the underlying effusion with sensitivity being higher for the larger effusion [18].

There was some evidence that clinical experience of the examiners may influence reliability and accuracy. Two studies [14], [18] suggested experienced clinicians achieved higher reliability than those which included less experienced assessors [15], [16]. One study reported on sensitivity and specificity for assessment of knee effusion for junior and more senior residents; although sensitivity was similar, specificity was higher for the senior resident [20]. These data suggest clinical tests of knee effusion like other physical examination techniques are to some extent dependent on clinical experience.

We had hoped at the outset to determine the optimum approach to clinical characterization of effusion in knee OA; however there were too few studies looking at validity in order to allow determination of the optimal test. A combination of clinical tests appeared to increase sensitivity in detecting effusion though with a corresponding reduction in specificity. Presently, therefore, for accurate identification as to the presence or absence of knee effusion imaging is needed. In the clinical setting where for example joint aspiration is being considered in the absence of imaging, it would appear sensible currently to undertake a combination of clinical tests to increase the likelihood of identifying an effusion.

In terms of reliability there was no good evidence that any one test is more reliable than any other—though head to head comparisons were difficult because of the marked difference in design and clinical setting of the studies in which the assessments were undertaken. Further studies using standardized method and comparing different tests are needed. At present where clinical assessment may be important in terms of predicting outcome then it would seem reasonable to suggest that formal assessment of reliability is undertaken in those studies.

Our focus in this review was studies which included subjects with knee OA. In other knee conditions including inflammatory arthritis, we found few studies that have looked at diagnostic accuracy and/or reliability of clinical assessments of knee effusion. In one study of patients with rheumatoid arthritis, the presence of knee effusion assessed by either the presence of fluctuant fluid in the medial or lateral knee compartment or through the presence of patellar tap, was compared with ultrasound assessment; the sensitivity of the clinical assessment was 59% and the specificity was 65% [25]. In another study, the ballottement test was used to assess for the presence of knee effusion following trauma; when compared against MRI effusion the test had a sensitivity of 83% and a specificity of 49% [26]. As for reliability, fluctuation test and patellar tap test when performed on 35 individuals with unilateral knee dysfunction had fair inter-observer agreement of κ = 0.37 and κ = 0.21, respectively [27]; however, intra-observer reliability was not assessed in this study.

Our review had several limitations. Many reviewed studies despite scoring well on COSMIN/CEBM had inadequate data and lacked elaboration on how the tests were performed including the scoring system and scales used thereby preventing pooling of their results. Reliability and diagnostic studies are often inadequately indexed in electronic databases and reliability data may also be embedded within other studies and as such not listed within key indexing terms making it difficult to retrieve all published articles. Given this, it is possible that despite an extensive and systematic search we may have missed some publications. Most of the studies included in this review had small samples and used different methods of assessment of reliability including kappa, weighted kappa (ordinal variables) and also reliability coefficients (continuous data) which are not directly comparable and so some caution is needed in interpreting the results. In one study too, the method used to generate the kappa was not described [16]. There were also only four studies that assessed the diagnostic quality of clinical examination of knee joint effusion against imaging such as US; however, only one evaluated a specific dynamic clinical test, that is, ballottement but gave little description on how this test was executed thereby limiting the generalizability of the findings. There was only one study that compared clinical assessment against MRI as the gold standard though there was insufficient data provided for it to be included in our review [22]. Also only a few studies described blinding of observers during reliability testing [12], [14], [20]. Several studies too were unclear with the time intervals between each set of measurements making it difficult to ascertain if the observed variability may in part be related to true subject change as in the study of Wood et al. [12] where the intra-observer reliability assessments were performed a month apart.

## Conclusion

Despite a variety of clinical tests to assess joint effusion in knee OA, the majority of unstandardized tests had relatively low intra- and inter-observer reliability. Reliability and diagnostic accuracy seems to be improved with experience. Compared to individual tests, using a combination of tests for effusion appears to improve sensitivity. Currently there is insufficient evidence to recommend any particular test in clinical practice.

## Figures and Tables

**Table 1:** Inter- and intra-observer reliability of assessment of knee effusion

References	Sample	Sample size	No. of observers, time interval between assessments	Clinical tests	Reliability values		
κ coefficient	95% CI	% Agreement
(A) *Inter-observer reliability of assessment of knee effusion*							
Cibere et al. [14]	OA	6	6 Experienced rheumatologists, same day. Pre- and post-standardization—results presented are post-standardization	Bulge sign	0.97^d^	–	–
Balloon test	0.82	–	–
Patellar tap	0.75	–	–

Cushnaghan et al. [15]	OA	8	5 Observers (2 consultants,3 trainees), within 3 h	aNon-bony swelling	0.28	0.12–0.44	–
Dervin et al. [16]	OA	115	6 Clinical fellows vs. 7 orthopedic staff, 7–10 days	aVisible Effusion	0.28	0.10–0.46	72
aBallottement	0.19	0.02–0.36	62
aSweep (bulge sign)	0.14	−0.04–0.32	58
Hart et al. [17]	Population sample including OA	41	2 Observers	aSoft Tissue swelling	0.25	–	97.5
Hauzeuer et al. [18]	Knee pain including OA/IA	11 (22 knees)	2 Skilled rheumatologists	Inspection (with other tests)	0.45^b^	0.04–0.85	–
Jones et al. [19]	OA	49 (98 knees)	5 Observers (consultant rheumatologist, consultant geriatrician, 2 rheumatology senior registrars, general medical registrar)	aJoint effusion (scale of 0-3 where: 0 = no effusion,1 = positive “bulge,” 2 = moderate effusion, 3 = tense effusion)	0.18	0–0.41	–
–	–
0.22
aJoint effusion (dichotomous)
Ulasli et al. [20]	OA	86 (172 knees)	2 Residents (2 and 4 years’ experience)	Bulge sign, ballottement, patellar tap	0.25	–	–
Wood et al. [12]	Recent presentation to primary care or to rheumatology clinics with knee problems that include OA or on waiting list for total knee replacement	58	2 Out of 3 physiotherapists, 5 minutes apart	Observation of swelling:			
medial gutter	0.28	–	71.9
suprapatellar	0.13	–	57.9
prepatellar	−0.02	–	94.6
infrapatellar	0.65	–	96.5
Bulge sign	−0.04	–	57.9
Balloon sign	−0.11	–	59.7
Patellar tap	−0.02	–	96.5
Judgment of effusion (dichotomous)	0.39	–	69.6
Judgment of effusion (4-point scale)	0.43^b^	–	–

(B) *Intra-observer reliability of assessment of knee effusion*							
Cushnaghan et al. [15]	OA	8	2 Consultants and 3 trainees, at least 30 min and several other patients apart	aNon-bony swelling	0.67^c^	0.45–0.89	–
Jones et al. [19]	OA	49 (98 knees)	5 Observers (consultant rheumatologist, consultant geriatrician, 2 rheumatology senior registrars, general medical registrar), within 3 h and after examining other patients in the intervening time	aJoint effusion (scale of 0-3 where: 0 = no effusion,1 = positive “bulge,” 2 = moderate effusion, 3 = tense effusion)	0.78^c^	0.63–0.93	–
–
–
0.91
aJoint effusion (dichotomous)
Wood et al. [12]	Recent presentation to primary care or to rheumatology clinics with knee problems that include OA or on waiting list for total knee replacement	58	1 of 3 physiotherapists, 1 month apart	Observation of swelling:			
medial gutter	0.57	–	85.1
suprapatellar	0.37	–	70.2
prepatellar	1	–	100.0
infrapatellar	0.66	–	97.9
Bulge sign	0.47	–	78.7
Balloon sign	0.37	–	79.2
Patellar tap	−0.03	–	93.6
Judgment of effusion (dichotomous)	0.32	–	66.0
Judgment of effusion (4 point scale)	0.35^b^	–	–

Abbreviations: OA, osteoarthritis; IA, inflammatory arthritis; CI, confidence interval; κ, kappa.

a Methods for assessment were not specified.

b Weighted kappa.

c Average.

d Reliability coefficient.

**Table 2:** Sensitivity and specificity of clinical assessments of knee effusion

References	Sample	Sample size	Assessment	% Sensitivity	% Specificity
D׳Agostino et al. [11]	OA	600	aSwelling (4-point scale) vs. US (moderate-important vs absent-minimal)	42.5	72.5
Esen et al. [21]	OA	100	Inspection (suprapatellar swelling) vs. US	32.7	88. 9
Ballottement vs. US	18.2	93.3
Hauzeur et al. [18]	OA/IA	50 (82 knees)	Inspection (with other tests) vs. US	Moderate effusion: 79	Moderate effusion: 25
Large effusion: 100	Large effusion: 78
Ulasli et al. [20]	OA	86 (172 knees)	Bulge sign, ballottement, patellar tap vs. US	Junior resident: 82.7	Junior resident: 35.3
Senior resident: 85.7	Senior resident: 47.2

Abbreviations: OA, osteoarthritis; IA, inflammatory arthritis; US, ultrasound.

a Clinical test/s not specified
