# Supplementary material for: Clinical assessment of effusion in knee osteoarthritis—A systematic review
Source: Semin Arthritis Rheum. 2016 Apr;45(5):556–63. doi: 10.1016/j.semarthrit.2015.10.004 (PMC4823277; doi:10.1016/j.semarthrit.2015.10.004)
Supplement: Supplementary file 1 — Supplementary Material [file mmc1.docx]

**Appendix 1 (online) : MEDLINE search strategy**

1   *Osteoarthritis, Knee/ or *Knee/ or *Knee Joint/               
2   limit 1 to (full text and humans)                              
3   Osteoarthritis/ or *Osteoarthritis, Knee/                      
4   limit 3 to (full text and humans)                              
5   osteoarthrosis.mp. or *Osteoarthritis/                        
6   limit 5 to (full text and humans)                               
7   4 or 6                                                         
8   1 and 7                                                         
9   *Knee Joint/ or *Osteoarthritis, Knee/                         
10   limit 9 to (full text and humans)                              
11   8 or 10                                                        
12   *Synovitis/ or *Knee Joint/ or *Diagnosis, Differential/       
13   limit 12 to (full text and humans)                             
14   *Knee Joint/ or *Synovial Fluid/ or joint effusion.mp.         
15   limit 14 to (full text and humans)                             
16   13 or 15                                                       
17   clinical test.mp. or *Physical Examination/                    
18   limit 17 to (full text and humans)                              
19   *Physical Examination/cl, is, mt, st [Classification, Instrumentation, Methods, Standards]             
20   limit 19 to (full text and humans)                              
21   *Symptom Assessment/is, mt, st [Instrumentation, Methods, Standards]    
22   limit 21 to (full text and humans)                               
23   18 or 20 or 22                                                  
24   16 and 23                                                         
25   *Magnetic Resonance Imaging/cl, is, mt, st [Classification, Instrumentation, Methods, Standards]   
26   limit 25 to (full text and humans)                             
27   *Magnetic Resonance Imaging/ or *Tomography, X-Ray Computed/ or *Radionuclide Imaging/
28   limit 27 to (full text and humans)                             
29   *Ultrasonography/cl, is, mt, st [Classification, Instrumentation, Methods, Standards]  
30   limit 29 to (full text and humans)                              
31   26 or 28 or 30                                                 
32   24 or 31                                                       
33   11 and 32
